# Supplementary material for: Size-Dependent Scaling of Stingless Bee Flight Metabolism Reveals an Energetic Benefit to Small Body Size
Source: Integr Comp Biol. 2022 Sep 6;62(5):1429–38. doi: 10.1093/icb/icac131 (PMC9825317; doi:10.1093/icb/icac131)
Supplement: icac131_Supplemental_File [file icac131_supplemental_file.docx]

**Supplmentary table**

**Table 1.** Insects used to compare flight metabolic rates across flying insect species in order of classification with masses, flight metabolic rates, and references used for mining the data.

| Order | Family | Species | References | Mass (g) | Flight Met. Rate (w) |
| --- | --- | --- | --- | --- | --- |
| Coleoptera | Cerambycidae | *Phorocantha semipunctata* | Chappel & Rogowitz 2000 | 0.3150 | 0.0245 |
| Coleoptera | Scarabaeidae | *Cotinus mutabilis* | Josephson *et al.* 2001 | 1.2000 | 0.8639 |
| Coleoptera | Scarabaeidae | *Cotinus texana* | Chappell 1984 | 1.2900 | 0.4450 |
| Coleoptera | Scarabaeidae | *Mecynorrhina savagei* | Klok, J. unpublished | 5.5063 | 0.8918 |
| Coleoptera | Scarabaeidae | *Pachnoda sinuata* | Auerswald et al. 1998 | 1.0000 | 0.0074 |
| Dictyoptera | Blattidae | *Periplaneta americana* | Niven Scharlemann 2005' | 1.2053 | 0.1527 |
| Diptera | Calliphoridae | *Lucilia sericata* | Niven Scharlemann 2005' | 0.0326 | 0.0134 |
| Diptera | Culicidae | *Aedes flavescens* | Niven Scharlemann 2005' | 0.0032 | 0.0006 |
| Diptera | Culicidae | *Aedes nearcticus* | Niven Scharlemann 2005' | 0.0058 | 0.0011 |
| Diptera | Drosophilidae | *Drosophila melanogaster* | Heymann & Lehmann 2006, Lehmann & Schutzner 2009, Lehmann 2001, Niven & Scharlemann 2005 | 0.0010 | 0.0001 |
| Diptera | Drosophilidae | *Drosophila mimica* | Niven & Scharlemann 2005' | 0.0028 | 0.0004 |
| Diptera | Drosophilidae | *Drosophila nikananu* | Niven & Scharlemann 2005' | 0.0006 | 0.0001 |
| Diptera | Drosophilidae | *Drosophila virilis* | Niven & Scharlemann 2005' | 0.0014 | 0.0001 |
| Hemiptera | Cicadidae | *Fidicina mannifera* | Bartholomew & Barnhart 1984 | 2.8380 | 0.6471 |
| Hymenoptera | Apidae | *Apis mellifera* | Niven & Scharlemann 2005' | 0.0979 | 0.0252 |
| Hymenoptera | Apidae | *Bombus edwardsii* | Niven & Scharlemann 2005' | 0.4000 | 0.1832 |
| Hymenoptera | Apidae | *Bombus lucorum* | Niven & Scharlemann 2005' | 0.5113 | 0.0913 |
| Hymenoptera | Apidae | *Bombus terrestris* | Darveau *et al.* 2014,  Hedenstrom *et al.* 2001 | 0.1678 | 0.0916 |
| Hymenoptera | Apidae | *Eufriesa spp.* | Darvaeau et al. 2005' | 0.4000 | 0.1644 |
| Hymenoptera | Apidae | *Eufriesia pulchra* | Casey *et al.* 1985,  Darvaeau *et al.* 2005' | 0.3879 | 0.2036 |
| Hymenoptera | Apidae | *Euglossa bursigera.* | Darvaeau *et al.* 2005' | 0.0840 | 0.0473 |
| Hymenoptera | Apidae | *Euglossa championi* | Darvaeau *et al.* 2005' | 0.1360 | 0.0519 |
| Hymenoptera | Apidae | *Euglossa cognata* | Darvaeau *et al.* 2005' | 0.1590 | 0.0831 |
| Hymenoptera | Apidae | *Euglossa crassipunctata* | Darvaeau *et al.* 2005' | 0.0670 | 0.0315 |
| Hymenoptera | Apidae | *Euglossa despecta* | Darvaeau *et al.* 2005' | 0.1120 | 0.0572 |
| Hymenoptera | Apidae | *Euglossa dissimula* | Casey *et al.* 1985,  Darvaeau *et al.* 2005' | 0.1020 | 0.0625 |
| Hymenoptera | Apidae | *Euglossa hansoni* | Darvaeau *et al.* 2005' | 0.0820 | 0.0549 |
| Hymenoptera | Apidae | *Euglossa heterosticta* | Darvaeau *et al.* 2005' | 0.0640 | 0.0357 |
| Hymenoptera | Apidae | *Euglossa imperialis* | Casey *et al.* 1985,  Darvaeau *et al.* 2005' | 0.1727 | 0.0974 |
| Hymenoptera | Apidae | *Euglossa mandibularis* | Casey *et al.* 1985 | 0.9025 | 0.0986 |
| Hymenoptera | Apidae | *Euglossa mixta* | Darvaeau *et al.* 2005' | 0.0940 | 0.0568 |
| Hymenoptera | Apidae | *Euglossa saphirina* | Casey *et al.* 1985,  Darvaeau *et al.* 2005' | 0.0630 | 0.0409 |
| Hymenoptera | Apidae | *Euglossa spp.* | Darvaeau *et al.* 2005' | 0.1000 | 0.0587 |
| Hymenoptera | Apidae | *Euglossa tridentata.* | Darvaeau *et al.* 2005' | 0.1100 | 0.0652 |
| Hymenoptera | Apidae | *Eulaema bombiformis* | Darvaeau *et al.* 2005' | 0.9830 | 0.4847 |
| Hymenoptera | Apidae | *Eulaema cingulata* | Casey *et al.* 1985,  Darvaeau *et al.* 2005' | 0.5454 | 0.2323 |
| Hymenoptera | Apidae | *Eulaema meriana* | Casey *et al.* 1985,  Darvaeau *et al.* 2005' | 0.9077 | 0.2876 |
| Hymenoptera | Apidae | *Eulaema nigrita* | Casey *et al.* 1985,  Darvaeau *et al.* 2005' | 0.4198 | 0.2217 |
| Hymenoptera | Apidae | *Eulaema spp.* | Darvaeau *et al.* 2005' | 0.8000 | 0.1761 |
| Hymenoptera | Apidae | *Exaerete frontalis* | Casey *et al.* 1985,  Darvaeau *et al.* 2005' | 0.6716 | 0.2023 |
| Hymenoptera | Apidae | *Exaerete spp.* | Darvaeau *et al.* 2005' | 0.8000 | 0.1526 |
| Hymenoptera | Apidae | *Frieseomelitta nigra* | (this publication) | 0.0108 | 0.0023 |
| Hymenoptera | Apidae | *Lestrimelitta danuncia* | (this publication) | 0.0096 | 0.0020 |
| Hymenoptera | Apidae | *Melipona panamica* | (this publication) | 0.0734 | 0.0323 |
| Hymenoptera | Apidae | *Melipona triplaridis* | (this publication) | 0.1157 | 0.0483 |
| Hymenoptera | Apidae | *Plebeia franki* | (this publication) | 0.0027 | 0.0006 |
| Hymenoptera | Apidae | *Plebeia frontalis* | (this publication) | 0.0037 | 0.0007 |
| Hymenoptera | Apidae | *Scaptotrigona lutipinnis* | (this publication) | 0.0144 | 0.0037 |
| Hymenoptera | Apidae | *Scaptotrigona panamensis* | (this publication) | 0.0141 | 0.0037 |
| Hymenoptera | Apidae | *Tetragonisca angustula* | (this publication) | 0.0047 | 0.0012 |
| Hymenoptera | Apidae | *Trigona fulviventris* | (this publication) | 0.0168 | 0.0032 |
| Hymenoptera | Apidae | *Trigona muzoensis* | (this publication) | 0.0117 | 0.0016 |
| Hymenoptera | Apidae | *Trigonosca atomaria* | (this publication) | 0.0018 | 0.0003 |
| Hymenoptera | Apidae | *Trigonosca bouyssoni* | (this publication) | 0.0015 | 0.0001 |
| Hymenoptera | Apidae | *Xylocopa californica* | Niven & Scharlemann 2005' | 0.6000 | 0.2219 |
| Hymenoptera | Apidae | *Xylocopa capensis* | Niven & Scharlemann 2005' | 1.2000 | 0.3734 |
| Hymenoptera | Megachilidae | *Megachile rotundata* | Bennett *et al.* 2013, 2014 | 0.0335 | 0.0025 |
| Hymenoptera | Pteromalidae | *Nasonia giraulti* | Lehmann & Heymann 2006' | 0.0004 | 0.0003 |
| Hymenoptera | Pteromalidae | *Nasonia longicornis* | Lehmann & Heymann 2006' | 0.0006 | 0.0003 |
| Hymenoptera | Pteromalidae | *Nasonia vitripennis* | Lehmann & Heymann 2006' | 0.0005 | 0.0004 |
| Lepidoptera | Lasiocampidae | *Artace sp.* | Bartholomew & Casey 1978 | 0.1286 | 0.0256 |
| Lepidoptera | Lasiocampidae | *Odonestis pruni* | Niven & Scharlemann 2005' | 0.2550 | 0.0473 |
| Lepidoptera | Megalpygidae | *Megalpyge sp.* | Bartholomew & Casey 1978 | 0.6270 | 0.1922 |
| Lepidoptera | Noctuidae | *Agrotis exclamationis* | Niven & Scharlemann 2005' | 0.2000 | 0.0465 |
| Lepidoptera | Noctuidae | *Agrotis pronuba* | Niven & Scharlemann 2005' | 0.2733 | 0.6305 |
| Lepidoptera | Noctuidae | *Cucullia lactucae* | Niven & Scharlemann 2005' | 0.2850 | 0.0444 |
| Lepidoptera | Noctuidae | *Plusia gamma* | Niven & Scharlemann 2005' | 0.1200 | 0.0261 |
| Lepidoptera | Notodontidae | *Apetaloides firmiana* | Bartholomew & Casey 1978 | 0.1690 | 0.0602 |
| Lepidoptera | Nymphalidae | *Melitaea cinxia* | Niitepold & Hanski 2013 | 0.1000 | 0.0056 |
| Lepidoptera | Nymphalidae | *Vanessa io* | Niven & Scharlemann 2005' | 0.2044 | 0.0286 |
| Lepidoptera | Nymphalidae | *Vanessa polychloros* | Niven & Scharlemann 2005' | 0.2700 | 0.1052 |
| Lepidoptera | Saturniidae | *Adeloneivaia boisduvalii* | Bartholomew & Casey 1978 | 0.9363 | 0.1091 |
| Lepidoptera | Saturniidae | *Adeloneivaia subungulata* | Bartholomew & Casey 1978 | 0.4870 | 0.2092 |
| Lepidoptera | Saturniidae | *Aglia tau* | Niven Scharlemann 2005' | 0.1125 | 0.0443 |
| Lepidoptera | Saturniidae | *Antheraea pernyi* | Niven & Scharlemann 2005' | 0.8297 | 0.0495 |
| Lepidoptera | Saturniidae | *Automerina auletes* | Bartholomew & Casey 1978 | 0.7200 | 0.3459 |
| Lepidoptera | Saturniidae | *Automeris fieldi* | Bartholomew & Casey 1978 | 0.3940 | 0.0998 |
| Lepidoptera | Saturniidae | *Automeris hamata* | Bartholomew & Casey 1978 | 0.5640 | 0.1687 |
| Lepidoptera | Saturniidae | *Automeris jacunda* | Bartholomew & Casey 1978 | 0.5991 | 0.0795 |
| Lepidoptera | Saturniidae | *Automeris zugana* | Bartholomew & Casey 1978 | 0.5523 | 0.0760 |
| Lepidoptera | Saturniidae | *Dirphea agis* | Bartholomew & Casey 1978 | 0.1970 | 0.1892 |
| Lepidoptera | Saturniidae | *Eacles imperialis* | Bartholomew & Casey 1978 | 1.1050 | 0.2742 |
| Lepidoptera | Saturniidae | *Hyperchirica nausica* | Bartholomew & Casey 1978 | 0.2160 | 0.1053 |
| Lepidoptera | Saturniidae | *Saturnia pavonia* | Niven & Scharlemann 2005' | 0.1983 | 0.0951 |
| Lepidoptera | Saturniidae | *Sphingicampa quadrilineata* | Bartholomew & Casey 1978 | 0.8180 | 0.2288 |
| Lepidoptera | Saturniidae | *Syssphinx molina* | Bartholomew & Casey 1978 | 1.7570 | 0.3631 |
| Lepidoptera | Sphingidae | *Deilephila elpenor* | Niven & Scharlemann 2005' | 0.6500 | 0.2178 |
| Lepidoptera | Sphingidae | *Enyo ocypete* | Bartholomew & Casey 1978 | 0.4145 | 0.2114 |
| Lepidoptera | Sphingidae | *Erinnyis ello* | Bartholomew & Casey 1978 | 1.2100 | 0.3202 |
| Lepidoptera | Sphingidae | *Hyles euphorbia* | Niven & Scharlemann 2005' | 0.6500 | 0.2028 |
| Lepidoptera | Sphingidae | *Madoryx oeclus* | Bartholomew & Casey 1978 | 1.6990 | 0.6652 |
| Lepidoptera | Sphingidae | *Manduca corallina* | Bartholomew & Casey 1978 | 1.6183 | 0.0030 |
| Lepidoptera | Sphingidae | *Manduca corallina* | Bartholomew & Casey 1978 | 1.6183 | 0.5906 |
| Lepidoptera | Sphingidae | *Manduca lefeburei* | Bartholomew & Casey 1978 | 0.5710 | 0.2410 |
| Lepidoptera | Sphingidae | *Manduca rustica* | Bartholomew & Casey 1978 | 2.8100 | 0.8009 |
| Lepidoptera | Sphingidae | *Oryba achemenides* | Bartholomew & Casey 1978 | 2.8085 | 1.1960 |
| Lepidoptera | Sphingidae | *Pachygonia drucei* | Bartholomew & Casey 1978 | 0.7020 | 0.3605 |
| Lepidoptera | Sphingidae | *Pachylia ficus* | Bartholomew & Casey 1978 | 3.2250 | 1.0912 |
| Lepidoptera | Sphingidae | *Perigonia lusca* | Bartholomew & Casey 1978 | 0.5583 | 0.2796 |
| Lepidoptera | Sphingidae | *Protambulyx strigilis* | Bartholomew & Casey 1978 | 1.1097 | 0.1730 |
| Lepidoptera | Sphingidae | *Xylophanes libya* | Bartholomew & Casey 1978 | 0.5590 | 0.2278 |
| Lepidoptera | Sphingidae | *Xylophanes pluto* | Bartholomew & Casey 1978 | 0.8280 | 0.3739 |
| Lepidoptera | Sphingidae | *Deilephila euphorbiae* | Niven & Scharlemann 2005' | 0.3950 | 0.1397 |
| Odonata | Aeshnidae | *Aeshna multicolor* | Henry & Harrison 2014' | 0.6338 | 0.0460 |
| Odonata | Aeshnidae | *Anax junius* | Henry & Harrison 2014' | 1.2329 | 0.1335 |
| Odonata | Libellulidae | *Libellula comanche* | Henry & Harrison 2014' | 0.3882 | 0.1586 |
| Odonata | Libellulidae | *Libellula luctuosa* | Henry & Harrison 2014' | 0.2847 | 0.0459 |
| Odonata | Libellulidae | *Libellula saturata* | Henry & Harrison 2014' | 0.4311 | 0.1591 |
| Odonata | Libellulidae | *Macrodiplax balteata* | Henry & Harrison 2014' | 0.2189 | 0.0765 |
| Odonata | Libellulidae | *Pachydiplax longipennis* | Henry & Harrison 2014' | 0.1631 | 0.0626 |
| Odonata | Libellulidae | *Pantala flavescens* | Henry & Harrison 2014' | 0.1496 | 0.0624 |
| Odonata | Libellulidae | *Pantala hymenaea* | Henry & Harrison 2014' | 0.2997 | 0.3824 |
| Odonata | Libellulidae | *Tramea lacerata* | Henry & Harrison 2014' | 0.4387 | 0.0975 |
| Odonata | Libellulidae | *Tramea onusta* | Henry & Harrison 2014' | 0.3534 | 0.0868 |
| Orthoptera | Acrididae | *Locusta migratoria* | Snellig *et al.* 2012 | 0.9630 | 0.1306 |
| Orthoptera | Acrididae | *Schistocerca americana* | Rascon Harrison 2005 | 1.2200 | 0.1530 |
| Orthoptera | Acrididae | *Schistocerca gregaria* | Niven & Scharlemann 2005' | 1.7365 | 0.0043 |
| Orthoptera | Acrididae | *Schistocerca gregaria* | Niven & Scharlemann 2005' | 1.9600 | 0.1207 |
